# Assessment of functional treatment versus plaster of Paris in the treatment of grade 1 and 2 lateral ankle sprains

**DOI:** 10.1007/s10195-014-0289-8

**Published:** 2014-03-27

**Authors:** Muhammad Naeem, Muhammad Kazim Rahimnajjad, Nasir Ali Rahimnajjad, Zaki Idrees, Ghazanfar Ali Shah, Ghulam Abbas

**Affiliations:** B-8, Akbar Apartments, Bleak House Road, Civil Lines, Cantt. Karachi, Karachi, 74200 Pakistan

**Keywords:** Ankle injury, Visual analog scale, Plaster of Paris

## Abstract

**Background:**

Despite the common occurrence of ankle sprains, no treatment is considered to be the gold standard for the management of such sprains. We assessed functional treatment versus plaster of Paris (POP) for the treatment of lateral ankle sprains, with pain and function employed as the outcome measures.

**Materials and methods:**

126 Patients were eligible for inclusion. They were assigned to either the functional treatment Tubigrip (TG) group or the POP group after applying block randomization. Characteristics such as age, dominant ankle, and gender were assessed at baseline. Pain and functional assessments were done using the visual analog scale (VAS) and the Karlsson score (KS) at baseline (at the start of the study) and during the 2nd and 6th weeks, respectively. Data on other subjective parameters, such as the number of painkillers used, the number of days taken off work, and the number of sleepless nights, were requested from the patients at the end of the study. SPSS version 16 was used for analysis, and *p* < 0.05 was taken to indicate significance.

**Results:**

60 Patients completed the trial in each group. The mean ages were 28.77 ± 6.72 in the TG group and 29.83 ± 6.30 in the POP group (*p* = 0.034). There was a slight female predominance. Right and left ankles were equally involved in the TG group, while left ankles were mainly involved in the POP group. Mean differences in VAS and KS between the two groups were statistically significant at the end of the study. The mean number of painkillers used by the patients in the TG group was higher than the number used in the POP group (*p* < 0.001). The mean number of days taken off work was 4.18 ± 1.73 days in the TG group, and 6.25 ± 2.73 days in the POP group (*p* < 0.001). The mean number of sleepless nights was higher in the POP group.

**Conclusion:**

The results of our study indicate that functional treatment provides better functional support and pain reduction than a below-knee POP cast.

**Level of evidence:**

Level I.

## Introduction

The recent emphasis of health professionals on physical fitness has resulted in decreases in morbidity and mortality but a rise in sports-related injuries [1, 2]. Ankle injuries, particularly ankle sprains, are the most common sports-related injuries, and are currently the reason for 3–5 % of all ER visits in the UK, and 10 % of them in the USA [3]. The mechanism of injury in a lateral ankle sprain may include inversion of the plantarflexed foot [2–4]. Crichton proposed the grading of these injuries based on ankle sprain severity [2].

The shibboleth RICE (rest, ice, compression, and elevation) is still used despite a lack of evidence for the benefit of compression. Conventional treatment includes early mobilization with weight bearing with or without the use of external support. External supports include tape, a brace, or elastic bandages, which are preferred to plaster of Paris (POP) because they gave better functional outcomes in previous studies [5]. A meta-analysis by Kerkhoffs and colleagues [6] showed no significant difference in the functional outcomes of cast immobilization and functional treatment. Contrary to the results obtained by Kerkhoffs, another study [7] provided evidence from a controlled clinical trial that a below-knee POP cast can increase the rate of healing of the sprained ankle, and they showed that cast immobilization was superior to other treatments during the first three months, but that with other functional treatments were equally effective after 9 months. Although no ankle sprain treatment is currently considered the gold standard, one survey found that functional treatment is being used by nearly 70 % of all doctors [8]. Studies also advocate that surgical management is a good option for sprains that result in instability, but this has not been shown to be superior over other treatments [9, 10].

Quality of life is another significant concern among these patients: while treatment may result in the resolution of symptoms, late manifestations in the form of recurrent sprains, pain, swelling, and instability may affect 30–50 % of such patients [2]. We hypothesized that functional treatment provides better resolution of pain and functionality than a below-knee POP cast; therefore, bearing in mind the treatment strategies currently being practiced, we assessed functional treatment versus POP for the treatment of lateral ankle sprains of grade 1 or 2, employing functionality and pain reduction as the outcomes of the study.

## Materials and methods

**Fig. 1:**
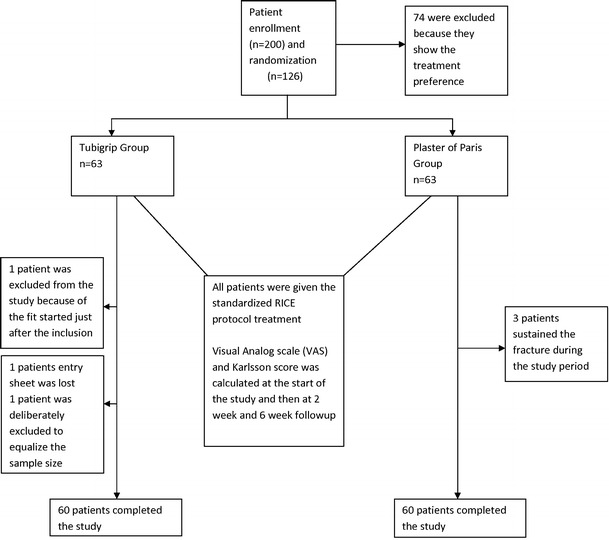
CONSORT diagram

This study was performed at the Department of Emergency and Trauma in a tertiary care teaching hospital from January 2011 until July 2011. During this period, 200 patients were enrolled after meeting the following inclusion criteria: grade 1 or 2 lateral ankle sprain (according to the Crichton classification, ankle sprains are grouped into three grades: I stretched ligaments (not torn), with a stable joint and a negative anterior drawer test; II partially torn ligament with a lax joint and a partially positive anterior drawer test; III complete ligament rupture with an unstable joint and a positive anterior drawer test [2]); <40 years of age for both sexes; presention within 48 h of injury. Emergency radiographs were taken of both anteroposterior and lateral views to rule out any fractures. Only patients >18 years were included. Patients aged <18 years; those who presented >48 h after the injury; those with fractures, multiple injuries, any neurological or musculoskeletal illness, or any comorbidity associated with long-term disability; and non-local residents were excluded from the study. All patients gave their informed consent prior to inclusion in the study. Only 126 patients agreed to participate in the study. After obtaining informed consent, the patients were randomized such that each patient was allocated to either a functional treatment (group A) or treatment with a plaster of Paris cast (group B). None of the patients opted for a particular treatment on their own; they were all assigned to the groups by the investigators using the block randomization technique. The standardized treatment based on the RICE (rest, ice, compression, and elevation) protocol was given to all patients. Either “TG” (denoting the functional treatment) or “POP” (for plaster of Paris) was written on each of 126 sheets of white paper which were placed in envelopes. The patients were allowed to pick the envelope of their choice. The authors were blinded until the opening of the envelopes by the patients. Each patient’s usage of analgesia (in the form of paracetamol) was specifically noted. The patients in the TG group received the functional treatment and the patients in the POP group were given a below-knee plaster cast. Patients were followed up at 2 weeks and again at 6 weeks. The Karlsson score was noted at the time of presentation, as well as at 2 and 6 weeks. Pain was scored on the visual analog scale (VAS) at the time of presentation and again at 2 and 6 weeks. The CONSORT diagram for patient participation in the trial is shown in Fig. 1.

All information was gathered using a structured questionnaire in which characteristics such as age, dominant ankle, and gender were assessed at baseline. Pain and functional assessments were done using the visual analog scale and Karlsson score, respectively, at baseline and in the 2nd and 6th weeks. Data on other subjective parameters such as the number of painkillers used, the number of days taken off work, and the number of sleepless nights were requested from the patients at the end of the study. All data were analyzed using the Statistical Package for Social Sciences software (SPSS), version 16.0. Student’s *t* test and chi square were used to assess the *p* value. *p* < 0.05 was taken to indicate significance. By setting Cohen’s *d* to 0.5, the probability value to 0.05, and the sample size to 120, the power (calculated after the completion of the trial) of the study was found to be 85.93 % for a one-tailed hypothesis.

## Results

Among the 120 patients who completed the study, 60 were assigned to the functional treatment group, and the other 60 to the POP group. The mean ± SD age of the patients in the TG group was 28.77 ± 6.72 years, and that of the patients in the POP group was 29.83 ± 6.30 years. The difference between the two groups was statistically significant (*p* = 0.034). Right and left ankles were equally affected in the TG group (i.e., there were 30 right and 30 left ankles that were affected), while the dominant ankle involved was the left one in 39 patients in the POP group and the right one in 21 patients (*p* < 0.001). There was a slight female predominance in both groups (i.e., 35 in the TG group and 42 in the POP group), while the remaining patients were male; the significance level of this female predominance was *p* < 0.001.

The mean visual analog scale score at presentation was 8.40 ± 0.92 in the TG group and 8.27 ± 0.94 in the POP group. This difference in the scores of the groups was statistically nonsignificant (*p* = 0.434). The mean visual analog scale score at 2 weeks was 6.15 ± 0.75 in the TG group and 6.28 ± 0.11 in the POP group. This difference in the scores of the groups was statistically nonsignificant (*p* = 0.376). The mean visual analog scale score at 6 weeks was 3.88 ± 0.85 in the TG group and 4.97 ± 0.82 in the POP group. This difference in the scores of the groups was statistically significant (*p* < 0.001). The mean Karlsson score at presentation was 21.17 ± 6.31 in the TG group and 23.67 ± 5.24 in the POP group; this difference in the scores of the groups had a statistical significance of *p* = 0.020. The mean Karlsson score at 2 weeks was 52.03 ± 6.47 in the TG group and 52.37 ± 5.33 in the POP group. This difference in the scores of the groups was statistically nonsignificant (*p* = 0.759). The mean Karlsson score at 6 weeks was 76.25 ± 10.67 in the TG group and 70.10 ± 6.35 in the POP group; the difference in the scores of the groups had a statistical significance of *p* < 0.001.

The mean number of painkillers used by the patients in the TG group was 6 ± 2.85, and it was 8 ± 2.58 in the POP group. The difference in the scores of the groups was statistically significant (*p* < 0.001). The mean number of days taken off from work was 4.18 ± 1.73 days in the TG group and 6.25 ± 2.73 days in the POP group (*p* < 0.001). The mean number of sleepless nights was 3.57 ± 1.56 days in the TG group and 5.45 ± 1.88 days in the POP group. The difference in the scores of the groups had a significant *p* value of <0.001. All of these results are presented in tabulated form in Tables 1 and 2.

**Table 1:** Pretreatment characteristics, visual analog scale scores, and Karlsson scores of the patients

Variable	TG	POP	*p* value
Age (years)	28.77 ± 6.72	29.83 ± 6.30	0.034*
Ankle			
Right	30	21	<0.001*
Left	30	39	
Sex			
Male	25	18	<0.001*
Female	35	42	
VAS at presentation	8.40 ± 0.92	8.27 ± 0.94	0.434
Karlsson score at presentation	21.17 ± 6.31	23.67 ± 5.24	0.571

*VAS* visual analog scale for pain, *TG* Tubigrip, *POP* plaster of Paris

* Denotes a statistically significant difference between the groups

**Table 2:** Post-treatment characteristics, visual analog scale scores, and Karlsson scores of the patients

Variable	TG	POP	*p* value
VAS at 2 weeks	6.15 ± 0.75	6.28 ± 0.11	0.376
VAS at 6 weeks	3.88 ± 0.85	4.97 ± 0.82	<0.001*
Karlsson score at 2 weeks	52.03 ± 6.47	52.37 ± 5.33	0.759
Karlsson score at 6 weeks	76.25 ± 10.67	70.10 ± 6.35	<0.001*
Number of painkillers	6 ± 2.85	8 ± 2.58	<0.001*
Number of days taken off work	4.18 ± 1.73	6.25 ± 2.73	<0.001*
Number of sleepless nights	3.57 ± 1.56	5.45 ± 1.88	<0.001*

*VAS* visual analog scale for pain, *TG* Tubigrip, *POP* plaster of Paris

* Denotes a statistically significant difference between the groups

## Discussion

The results of our study indicate that the functional treatment is a better treatment than a POP cast for lateral ankle sprains. We found that functional treatment provides better support in terms of pain reduction and provides more functional stability than a POP cast. Although many previous studies have compared one of the available treatments with another, none of them have provided sufficient evidence for the superiority of a particular treatment protocol, which may be the result of the use of poor study and assessment techniques. Cast immobilization, surgical repair, and functional treatments are considered the treatment options in the scientific literature [11, 12]. Cast immobilization utilizing a below-knee plaster cast is a double-edged sword, as it can help to speed up healing but can also result in functional impairment through muscle wasting (although evidence for such effects is lacking). A study by Lamb et al. [7] supports the usage of below-knee plaster cast immobilization. They found that functionality was improved at the 3-month interval when such a cast was used, but that all treatments (Aircast brace, Bledsoe boot, or 10-day below-knee cast and double-layer tubular compression bandage) were equally effective at the 9-month interval [7, 9, 10].

In our study, female patients were more affected. An epidemiological study from the United States [13] found that more sprains occurred between the ages of 10 and 19 years, while the mean affected age was higher in our study. The same study found that, among males, those between 15 and 24 years old were most commonly affected; among females, those >30 years old were most affected. Hosea and colleagues [14] found in their prospective study that grade 1 injuries were more prevalent among females, but the difference between the sexes was not statistically significant for grade 2 and 3 injuries. Both ankles were equally affected in the TG group, while left ankles were more likely to be affected in the POP group. Many studies have found that limb dominance does not manifest itself in ankle injuries, while one study found that limb dominance does indeed play a role in the injury mechanism [15].

The mean visual analog scale score was slightly higher in the TG group at the start of the study, while it was lower in the 2nd and 6th weeks. The difference between groups was statistically significant in the 6th week, showing that functional treatment is superior to POP in terms of pain reduction in patients with lateral ankle sprains. A prospective trial showed that patients treated with functional elastic wraps rather than cast immobilization experienced less pain in the third week (57 versus 87 %, *p* = 0.02) [16]. A meta-analysis showed that functional treatments provide better outcomes than immobilization when mild to moderate injuries were considered, but immobilization can speed up recovery from severe sprains [17].

The mean number of painkillers used by patients in the POP group was significantly higher than the mean number used by patients in the functional treatment group. One previous study found paracetamol to be just as effective as more potent NSAIDs at reducing pain [18]; considering the significant side effects of NSAIDs, paracetamol is therefore the better option. In our opinion, patients in the POP group may be more aware of their injuries, which was why more painkillers were consumed by that group. Hertel [19] found that painkillers along with the application of cold can reduce further injury caused by free radicals. These treatments can alleviate the pain and inflammation, but tissue repair requires a further period of 3 weeks. During this period, type III collagen replaces the type I collagen, increasing ligament strength, and the application of stress can aid proper fiber alignment. Other studies have also advocated proper muscular training after the injury [20, 21]. Since POP does not offer significant weight bearing, we believe that it may not aid proper healing.

As far as ankle rehabilitation is concerned, several studies have provided evidence that rehabilitation should improve the proprioception of the joints, thus reducing the rate of re-injury [22–24]. We found that the Karlsson scores were higher in the POP group at the start of the study, but they were significantly lower at the 6th week in the POP group when compared with the TG group. This might be because cast immobilization decreases functionality due to muscle wasting, although it contrasts with the findings of Lamb and colleagues [7], who advocated the use of a plaster cast due the resulting improved functional outcomes.

Our study found that the patients in the TG group required significantly fewer days off from work as compared to those in the POP group. Eiff and colleagues [16] also found that patients who underwent functional treatment returned to work earlier than those treated with cast immobilization (54 versus 13 %, *p* < 0.001). This indicates that ankle stability is not improved by immobilization. The management of acute sprains should consist of pain and swelling control along with range-of-motion exercises, including neuromuscular and strengthening exercises. Functionality is improved by early mobilization, and this leads to an earlier return to activities of daily life [25]. Many previous prospective studies have found that functional treatment is better at helping patients to maintain their mobility, which can strengthen the ankle joint and thus prevent further sprains [26–28]. Kerkhoffs and colleagues [29] found using an meta-analysis that functional treatment is superior to cast immobilization when treating sprains, but they further added that many of the trials reported so far were either of low quality or had high levels of bias.

Based on our results, and in the light of previous studies, we advocate that functional treatment provides better functional outcomes and pain reduction than POP. Given that its single-center setting is one of the main limitations of this study, a multi-center trial comparing the three treatment arms (functional treatment, plaster of Paris, and surgery) for all kinds of ankle sprains is needed. The other main limitation is the small observation period of this study, because we did not follow the patients beyond 6 weeks after the injury, which would have helped us to assess the long-term benefits of the treatments. At the end of the study, the patients were asked about their number of days off work, the number of painkillers they had used, and the number of sleepless nights they had endured, which may have a recall bias component. Finally, this was an unblinded study, which may also have influenced the outcomes.
